# Does the atrial incision affect rates of early postoperative atrial arrhythmias in mitral valve surgery?

**DOI:** 10.1093/icvts/ivad181

**Published:** 2023-11-07

**Authors:** Philippa Jane Temple Bowers, Stephanie Ch’ng

**Affiliations:** Wellington Regional Hospital, Wellington, New Zealand; Wellington Regional Hospital, Wellington, New Zealand

**Keywords:** review, mitral valve, atrial arrhythmia, incision

## Abstract

A best evidence topic in cardiac surgery was written according to a structured protocol. The question addressed was ‘in patients undergoing mitral valve surgery, does atrial incision affect early postoperative rates of atrial arrhythmia’. Two hundred and four papers were found. Nine represented the best evidence to answer the clinical question. The authors, journal, date and country of publication, patient group studied, study type, relevant outcomes and results of these papers are tabulated. Data suggest that a transeptal incision is associated with increased rates of postoperative atrial arrhythmia compared with direct left atriotomy.

## INTRODUCTION

A best evidence topic was constructed according to a structured protocol. This is fully described in the ICVTS [1].

## THREE-PART QUESTION

In [patients undergoing mitral valve surgery] does [atrial incision] affect [postoperative rates of atrial arrhythmia].

## CLINICAL SCENARIO

A 56-year-old male presents for mitral valve (MV) surgery with severe mitral regurgitation from ‘posterior leaflet’ prolapse. His left atrium is mildly dilated on transthoracic echocardiogram. He is in sinus rhythm (SR) with no history of atrial fibrillation (AF). Will the atrial incision affect the risk of early postoperative atrial arrhythmia (AA)?

## SEARCH STRATEGY

Medline 1950 to 2020 using Ovid interface:

[cardiac surgical procedures/OR heart atria/su [surgery] OR mitral valve insufficiency/su [surgery]]

AND [atrial fibrillation/ep, et [epidemiology, etiology] OR electrocardiography/OR postoperative complications/OR tachycardia, ectopic atrial/ep, et [epidemiology, etiology]]

AND [incision.mp OR trans$eptal.mp OR sondergaard$.mp OR interatrial groove.mp OR waterston$.mp OR superior trans$eptal.mp]

## SEARCH OUTCOME

Two hundred and four papers were found using the reported search. From these 9 papers were identified that provided the best evidence to answer the question. These are presented in Table 1. The search specifically focused on studies looking at patients undergoing MV surgery and early outcomes and excluded complex congenital surgery or atriotomy for other indications.

**Table 1:** Best Evidence Papers

Author, date, journal and country Study type (level of evidence)	Patient group	Outcomes	Key results	Comments
Utley *et al*. (1995), J Thorac Cardiovasc Surg, USA [2] Case series (IV)	149 patients undergoing MV surgery Group A: left atriotomy (*n* = 66) Group B: superior transeptal (*n* = 46) Group C: transeptal (*n* = 37)	AF (preoperative SR)	A (*n* = 6) B (*n* = 6) C (*n* = 0) Not significant (NS)	No statistically significant difference of postoperative rates of AF (in patients in SR preoperatively) noted between the 3 groups. Did not differentiate between AF and AFL.
Kumar *et al*. (1995), Ann Thorac Surg, Saudi Arabia [3] Case series (IV)	89 patients undergoing MV surgery Group A: left atriotomy (*n* = 24) Group B: superior transeptal (*n* = 65)	AF (24 h)	A: *n* = 5 B: *n* = 17	Did not differentiate between AF and AFL. No significant difference in rates of AF between groups. By 6 weeks follow-up most patients recovered their preoperative rhythms.
		AF (7 days)	A: *n* = 12 B: *n* = 25	
		AF (6 weeks)	A: *n* = 12 B: *n* = 26	
Bernstein *et al*. (1996), Pacing Clin Electrophysiol, USA [4] Case series (IV)	38 patients underwent MV surgery via superior transeptal approach	AF (with SR preoperatively)	*n* = 20	Did not differentiated between AF and AFL. Postoperative ECG 24–48 h post-operation, no patients in SR. Twenty patients were discharged home with atrial fibrillation or flutter. Transeptal approach improves exposure to mitral valve.
Masuda *et al*. (1996), Ann Thorac Surg, Japan [5] Cohort study (III)	152 patients who underwent MV surgery Group A: left atriotomy (*n* = 69) Group B: superior transeptal approach (*n* = 83)	AF (discharge)	A (*n* = 0) B (*n* = 2) NS	Although dysrhythmias tend to develop early in the postoperative period in Group B, reoperation was only significant predictor (*n* = 7; all of which had prolonged mitral disease). High incidence of early postoperative dysrhythmias is a reason for caution for use of superior transeptal approach. However, postoperative rhythms at the late follow-up were similar between the 2 groups. Follow-up ranged from 2 to 38 months. Did not differentiate between AF and AFL.
		AF (late)	A (*n* = 1) B (*n* = 2) NS	
Takeshita *et al*. (1997), Eur J Cardiothorac Surg, Japan [6] Case series (IV)	76 patients undergoing MV surgery Group A: left atriotomy (*n* = 22) Group B: superior transseptal (*n* = 54)	AF (of those in preoperative SR) A: 7 B: 19	A (*n* = 0) B (*n* = 2)	Sinus node function in Group B approach was relatively well maintained for more than 1 year after the operation. Only a small number of patients were in SR preoperatively [A: 7 (31.8%); B 19 (35.2%)]. Performed electrophysiological studies and angiography on all patients in sinus rhythm 12–16 months post-surgery (*n* = 9) which showed sinus node artery formally divided by incision. However, sinus node function was not severely impaired.
Gaudino *et al*. (1997), Ann Thorac Surg, Italy [7] Case series (IV)	146 patients undergoing MV surgery Group A: left atriotomy approach (*n* = 73) Group B: superior transeptal approach (*n* = 73)	AF (preoperative)	A: 57.5% B: 58.9% NS	No statistically significant difference in the cardiac rhythm in the early postoperative period was found between 2 groups among those patients in SR preoperatively. Prescence of preoperative SR only predictor of postoperative SR. Did not differentiate between AF and AFL. Type of atrial approach did not significantly affect late postoperative rhythm. Transection of sinus node artery and part of the internodal pathways leads to minor rhythm disturbances but do not seem to have major clinical implications. Long follow-up (25–27 months).
		AF (discharge)	A: 21.8% B: 54.6% NS	
		AF (late follow-up)	A: 28.1% B: 54% NS	
Tenpaku *et al*. (2000), Jpn J Thorac Cardiovasc Surg, Japan [8] Case series (IV)	95 patients undergoing MV surgery Group A: transseptal (T) (*n* = 40) Group B: superior transeptal (*n* = 33) Group C: left atriotomy (*n* = 22)	AF (in patients with preoperative SR)	A (*n* = 0) B (*n* = 0) C (*n* = 0)	Incidence of sinus node dysfunction statistically higher in the early postoperative period in Group B. However of the 40 patients in preoperative SR, 38 discharged home in SR. Did not differentiate between AF and AFL.
Nienaber and Glower (2006) Ann Thorac Surg, USA [9] Case series (IV)	531 patients undergoing MV surgery Group A: left atriotomy (*n* = 273) Group B: transeptal (*n* = 258)	AF	A (*n* = 69) B (*n* = 53) NS	Although more patients in Group B required pacemakers, incision type was NOT an independent predictor of this. Did not differentiate between AF and AFL. Single-centre, single surgeon study with no long-term follow-up.
Lukac *et al*. (2006) Ann Thorac Surg, Denmark [10] Cohort study (III)	213 patients undergoing MV surgery Group A: superior transeptal (*n* = 69) Group B: left atriotomy (*n* = 144)	AT	A (*n* = 24) B (*n* = 24) *P* 0.006	AT (sustained regular monomorphic atrial rhythm at a constant rate > 100 per minute—atrial flutter most frequent subgroup). Age >60, male sex and Group A predictors of AT. Only predictor of AF was age >60. AT was less frequent than AF in Group B, AT was as common as AF in Group A. Bedside telemetry for first 16–24 h followed by ECG if clinical suspicion of arrhythmia.
		AF	A (*n* = 31) B (*n* = 59) *P* 0.633	

AF, atrial fibrillation; AFL, atrial flutter; AT, atrial tachycardia; ECG, electrocardiogram; MV, mitral valve; SR, sinus rhythm.

## RESULTS

Utley *et al.* [2] prospectively looked at early postoperative outcomes in 149 patients undergoing MV surgery via left atriotomy, superior transeptal or transeptal incisions. There was no significant difference between groups with regards to AA and nodal rhythms. Permanent pacemaker (PPM) requirement was significantly higher in the superior transeptal group when compared to the left atriotomy group. The team concluded that there was a slight increase in risk of ‘loss of sinus rhythm’ in the superior transeptal incision when compared to the other incisions. Importantly, they did not differentiate between atrial tachycardia (AT) and AF. Of note, although the authors acknowledged the technical advantages of the superior transeptal approach affording an improved view of the MV, the majority of the MV repairs (a procedure usually demanding a quality view) were performed via a left atriotomy (27% via left atriotomy and 7% and 14% via superior transeptal and transeptal, respectively)—this may be more a reflection of surgeon preference, experience or valvular pathology at time of surgery (although aetiology appeared consistent between subgroups).

Kumar *et al.* [3] studied 89 patients undergoing MV surgery via left atriotomy (*n* = 25) and superior transeptal (*n* = 65) approaches. Three patients in the left atriotomy group developed a junctional rhythm, all which resolved on discharge. Twenty-five patients in the superior transeptal group developed a junctional rhythm, 3 remained in a junctional rhythm at 6 weeks. Again, the authors purported the advantages in view and visualization via the superior transeptal approach, however, as seen with in Utely *et al.*, rates of repair were actually lower in the superior transeptal group when compared to the atriotomy group. There was no mention of underlying aetiology of valve dysfunction to further comment.

Bernstein *et al.* [4] studied 38 patients who underwent MV surgery via a superior transeptal approach. Twenty patients were in AF or atrial flutter (AFl) on discharge with only 3% of those patients in SR preoperatively discharged in SR. At 6 months, 3 patients were in SR and the remainder remained in AF or low atrial activity. They concluded that the superior transeptal approach predictably led to ‘loss of the normal sinus mechanism’; however, there was no direct control or comparison group.

A retrospective study performed by Masuda *et al.* [5] in 1996 studied 152 consecutive patients undergoing MV surgery. Sixty-nine via left atriotomy and 83 via superior transeptal approaches. Twenty-four of 69 patients in the left atriotomy group were in SR preoperatively, of these, 8 patients experienced AA postoperatively with one patient discharged in AF. Nineteen of 83 patients in the superior transeptal group were in SR preoperatively. Ten of 18 (one mortality) patients experienced AA postoperatively and 4 were discharged with AA. This was not found to be statistically significant. The authors concluded that there was an observed increase in rates of arrhythmias immediately postoperatively in the superior transeptal group, these arrhythmias mostly resolved at follow-up and was similar to the left atriotomy group.

Takeshita *et al.* [6] studied 76 patients who underwent MV surgery via superior transeptal approach (*n* = 54) and left atriotomy (*n* = 22). They reported follow-up to 2 years. Nineteen patients in the superior transeptal group were in SR preoperatively. Of this group, 2 patients developed persistent longstanding postoperative AF. Seven patients in the left atriotomy group were in SR preoperatively with one patient developing a junctional rhythm requiring PPM. No patient remained in AF at 2 years. Takeshita *et al*. also performed preoperative and postoperative coronary angiograms on 9 patients undergoing superior transeptal approach during this study. In all 9 cases the SA nodal artery had been divided by the superior transeptal approach; however, nodal function ‘was not severely impaired’.

Gaudino *et al*. [7] set out to assess the safety and efficacy of the superior transeptal approach in 1997. They looked at 146 patients randomly assigned to undergo surgery via left atriotomy (73) or superior transeptal (73) incisions. Gaudino *et al*. reported no difference in rates of AA, including AF and junctional rhythms. They suggested preoperative factors such as left atrial (LA) dilatation, increased LA pressures and chronicity of mitral disease are more contributive to rates of postoperative rhythm disturbances than incision used. They did not differentiate between AFl and AF.

Tenpaku *et al.* [8] compared the effect of atrial incision on rates of postoperative arrhythmias in patients undergoing MV surgery. Forty patients with transeptal, 33 with superior transeptal and 22 patients with left atriotomy incisions. They noted that sinus node dysfunction and junctional rhythm were more common in the transeptal group. Maintenance of SR at mid-term follow-up was not statistically different between groups. Tenpaku *et al*. also assessed sinus nodal artery courses preoperatively in the superior transeptal group and found no clear correlation between course and postoperative sinus node dysfunction. They did demonstrate much higher rates of repair in the superior septal group than the other 2 incisions.

Nienaber and Glower [9] retrospectively reviewed 531 consecutive patients undergoing MV surgery. Left atriotomy was compared to the transeptal approach. Rates of AA was not significantly different. Rates of junctional rhythm and PPM requirement were high in the transeptal group. PPM requirement was attributed to increased complexity of surgery rather than the transeptal incision itself. This study did not differentiate between AT and AF. This was a single surgeon study, which limited surgical variance, and also was one of the largest study groups. The non-significant rates of arrhythmias may be related to the more conservative transeptal approach without superior extension which has been identified as likely cause of postoperatives ATs.

Lukac *et al.* [10] performed a retrospective study of 213 patients undergoing MV surgery via 3 different incisions to assess rates of ATs. It demonstrated that the superior transeptal approach conferred a higher risk of AA, most commonly AFl in the immediate postoperative period. They hypothesized that this was due to a long proarrhythmogenic posterior line of block. However, they did not demonstrate that the superior transeptal approach was an independent risk factor for AF. Of note, the superior transeptal approach was generally used in patients with smaller LA, which in turn is usually a sign of less chronic process of mitral disease and associated with lower rates of preoperative AF. They noted the importance of differentiating between AT (mainly AFl) and AF as pathophysiology and treatment differs. Study limitations include incomplete capture of AT episodes as monitoring not continuous.

## CLINICAL BOTTOM LINE

There remains a paucity of data looking at rates of AAs (particularly differentiating between AT/AFl and atrial fibrillation which is important as they differ in pathophysiology and also treatment) in relation to different access incisions in MV surgery. The majority of the studies are of low volume and difficult to directly compare due to differing study designs and outcome measures. The available data suggest that there is a higher risk of AAs when a transeptal approach (either superior or limited) is taken compared with a direct LA approach. It is felt that right atrial tachyarrhythmias are usually a result of the incision resulting in a proarrhythmogenic nidus whilst left AAs are due to the underlying pathology*.* Interestingly, although the vast majority of papers purport the advantage of the transeptal and superior transeptal approaches in regard to superior view of the MV, rates of repair via this superior incision were not reflected in all studies. Overall, the risk of re-entrant AAs needs to be weighed against the advantages of access to the MV achieved via transeptal approaches to the left atrium.

**Conflict of interest:** none declared.

## Data Availability

All relevant data are within the article and its supporting information files.
